# Navigating the Unknown: A Sensemaking Inquiry into the Mental Health Experiences of First-Year University Students at a Regional University in Fiji

**DOI:** 10.3390/bs16081424

**Published:** 2026-08-19

**Authors:** Kieran E. James, Sheikh A. Tanzil

**Affiliations:** 1 School of Business and Creative Industries, University of the West of Scotland, Paisley PA1 2BE, UK; 2 School of Accounting, Finance, and Economics, University of the South Pacific, Suva, Fiji; sheik.tanzil@usp.ac.fj

**Keywords:** first-year transition, mental health, Pacific Island students, regional university, Fiji, sensemaking

## Abstract

This study examines the mental health experiences of first-year university students at a regional university in Fiji that serves twelve Pacific Island nations. The study addresses a gap in Pacific higher education research, where student mental health has received limited systematic attention. This study uses an interpretive qualitative approach guided by sensemaking theory. It examines how first-year students understand the emotional, social, academic, and financial challenges of transitioning to university in a diverse Pacific higher education setting. Data was collected through three semi-structured group interviews with seventeen first-year students from different Pacific Island countries. Data was analysed using reflexive thematic analysis, supported by NVivo. Six interconnected themes were identified: homesickness and emotional separation; financial struggle and survival; academic pressure and feelings of inadequacy; identity reconstruction; social isolation and belonging; and resilience and coping. The findings show that students actively engage in sensemaking to interpret and manage disruptions to identity, relationships, and academic confidence. The study contributes to sensemaking theory by showing how it operates in a Pacific higher education context shaped by collective identity, cultural obligation, and, for many participants, Christian spiritual values.

## 1. Introduction

The relationship between the transition to higher education and student mental health has attracted sustained research attention in recent decades, particularly in Global North contexts where student wellbeing has become a central concern for institutional research and policy (Xiong et al., 2024; Naz et al., 2025). The first year of university is widely recognised as a period of heightened vulnerability. It is marked by identity disruption, social adjustment, and academic pressure in unfamiliar settings (Crookes, 2024; Lin et al., 2026). However, most of this evidence comes from Western and high-income university settings. The experiences of students in Pacific Island higher education contexts remain significantly underexplored (Chang, 2011; Crookes, 2021). This study addresses that gap. It focuses on first-year students at a regional university in Fiji that serves twelve Pacific Island nations.

The central research question guiding this study is: how do first-year students at a regional university in Fiji make sense of their mental health struggles during their transition to university?

The university studied here is not named in this article. It occupies a distinctive regional role, and identifying details are withheld because the institution is one of only a small number of comparable regional universities in the Pacific, which means that combining institutional identity with participant accounts could increase the risk of participants being indirectly identified within their small home communities. For context, the university was established in 1968 and is jointly owned by twelve Pacific Island nations, including Fiji, Samoa, Tonga, the Solomon Islands, Vanuatu, and Kiribati. Its main campus in Suva, Fiji, brings together students who often leave their home islands for the first time. For many, this transition is educational, social, and cultural. Students move from close-knit communities shaped by family networks and customary obligations into an urban academic setting that can feel unfamiliar (Cammock et al., 2021; Xiong et al., 2025).

The importance of the first-year transition is well established in higher education research, where it is linked to dropout risk and mental health decline (Gale & Parker, 2014; Tinto, 1993). In the Pacific Island context, these challenges are magnified by financial pressure, geographic separation from family support, limited access to mental health services, and strong community expectations placed on students as potential first-generation graduates (Charlson et al., 2019; Crookes, 2021; Naz et al., 2025). Homesickness in this setting extends beyond emotional distance. It also involves identity adjustment and the ongoing work of maintaining relational ties across large geographic and cultural distances (Xiong et al., 2025).

Despite the significance of these issues, qualitative research on Pacific Island student mental health in higher education remains limited (Chang et al., 2015; Crookes, 2024). Existing studies tend to focus on structural participation barriers or broad regional indicators rather than students’ subjective lived experiences (Hunter, 2012; Lin et al., 2026). We argue that qualitative inquiry is essential here because it captures how students interpret and give meaning to their ongoing struggles during transition.

Theoretically, we are guided by Weick’s (1995) theory of sensemaking. Sensemaking explains how individuals interpret ambiguous or disruptive situations by constructing meaning through experience, reflection, and social interaction. The first-year university transition represents precisely this kind of situation, particularly for students entering from Pacific Island contexts where academic and social environments may differ markedly from prior experience. Sensemaking offers a useful lens for understanding both the challenges students face and how they actively interpret and manage these challenges over time (Kowalenko et al., 2025; Upsher et al., 2025).

The article is structured as follows. Section 2 presents a thematic literature review covering student mental health, first-year transition, Pacific higher education, and sensemaking theory. Section 3 outlines the interpretive qualitative methodology. Section 4 presents six themes developed from the data. Section 5 concludes with key findings, implications, limitations, and directions for future research.

## 2. Literature Review

### 2.1. The Global Turn Towards the Study of Student Mental Health in Higher Education

Over the past decade, student mental health has become a major focus in higher education research and policy, particularly in the UK, Australia, the United States, and Canada. Large-scale studies consistently show rising levels of anxiety, depression, and psychological distress among university students, with first-year students identified as a particularly vulnerable group (Upsher et al., 2025; Xiong et al., 2024, 2025). Universities have responded by expanding counselling services, wellbeing initiatives, and peer support programmes, although questions remain about their effectiveness and cultural fit (Charlson et al., 2019; Naz et al., 2025).

At the same time, this literature is shaped largely by European and North American knowledge and institutional contexts. It tends to assume relatively smooth transitions from secondary to tertiary education, stable access to mental health services, and familiarity with university culture. These assumptions do not always hold in low-resource, geographically dispersed contexts such as the Pacific Islands, where educational preparation and support systems vary considerably (Chang, 2011; Crookes, 2024; Lin et al., 2026). This mismatch limits the direct transferability of Global North frameworks and points to the need for context-specific evidence rather than general claims about “the Global North,” which is itself a heterogeneous category.

### 2.2. First-Year Transition as a Complex Social Process

First-year transition has traditionally been explained through models of student integration. Tinto’s (1993) framework, for example, suggests that academic and social integration are key to student retention and success. This model has been widely criticised, however, for overemphasising individual adaptation while underplaying structural inequality and cultural differences (Gale & Parker, 2014). Students facing financial hardship, family obligations, or first-generation university status often encounter transition difficulties that integration models alone cannot fully explain (Crookes, 2021). Evidence from international student populations also shows that financial stress compounds the difficulties of educational transitions (Naz et al., 2025).

More recent scholarship reframes transition as a dynamic, multidimensional process involving identity change, emotional adjustment, and shifting social relationships (Gale & Parker, 2014; Upsher et al., 2025). Students are not simply adjusting to university; they are actively reconstructing who they are in relation to new academic and social contexts. This is particularly significant in Pacific Island settings, where students often move from collectivist home and village environments into institutions that emphasise individual performance and independence (Cammock et al., 2021; Crookes, 2024). This cultural mismatch is a key, and still under-examined, dimension of transition research. We note, however, that the twelve member nations of the university differ considerably from one another in language, social structure, and religious life, and the mismatch described here should be read as a general tendency rather than a uniform experience.

### 2.3. Pacific Higher Education and Structural Focus in Existing Research

Pacific Island higher education research has expanded in recent years but remains limited compared with global scholarship (Chang et al., 2015; Crookes, 2021; Lin et al., 2026). Much of this work focuses on access, participation, and structural barriers such as cost, geographic isolation, and shortages of teaching resources. These studies are important for highlighting regional inequality, but they tend to describe students through system-level constraints rather than lived struggles (Deva, 2014; Hunter, 2012).

As a result, there is limited understanding of how Pacific Island students experience and interpret higher education transition. Emerging work grounded in Pacific epistemologies argues for more relational and culturally grounded approaches to student wellbeing, emphasising community, spirituality, and collective identity (Cammock et al., 2021; Crookes, 2024). These perspectives remain underrepresented in mainstream higher education research, however, and there is still limited empirical work connecting these cultural frameworks to student mental health in a systematic way (Chang, 2011; Naz et al., 2025). This leaves a gap between structural descriptions of disadvantages and the realities of students navigating these conditions.

### 2.4. Homesickness, Identity Disruption, and Relational Distance

Homesickness is a well-known problem in the transition literature and is associated with emotional distress, anxiety, and reduced wellbeing (Gale & Parker, 2014; Xiong et al., 2025). It involves more than longing for home; it also disrupts identity and belonging. Students often struggle to maintain continuity between their pre-university and university selves (Crookes, 2024).

For Pacific Island students at a regional university in Fiji, these challenges are intensified by geographic distance, limited financial resources, and reduced access to communication with family and community networks (Cammock et al., 2021; Crookes, 2021; Lin et al., 2026). Unlike many international students in high-income countries, Pacific Island students often lack stable technological and financial support, which further complicates their ability to maintain relational ties (Xiong et al., 2025). For many participants, identity is closely tied to family and village membership, and relocation can disrupt these sources of belonging (Cammock et al., 2021). We note that the strength and form of this relational identity vary across the region’s diverse societies, and we do not treat it as a fixed or universal feature of Pacific culture. Homesickness in this context is therefore both emotional and structural, involving a real disruption to social meaning and belonging.

### 2.5. Financial Stress and Academic Pressure as Interlinked Challenges

Financial stress is consistently identified as a major predictor of poor mental health among university students (Charlson et al., 2019). Research on South Asian international students in high-income settings similarly shows how financial pressure compounds mental health struggles during educational transitions (Naz et al., 2025). In Pacific higher education contexts, financial pressure is often severe because of limited scholarship support and high living costs in urban centres such as Suva (Crookes, 2021). Students frequently report food insecurity, overcrowded housing, and difficulty affording academic materials. These conditions create ongoing psychological strain that extends beyond short-term financial worry (Crookes, 2024).

Financial stress is also tied closely to social and cultural expectations. Many students feel a strong sense of moral obligation towards their families and communities, particularly when they are the first in their family to attend university (Xiong et al., 2024, 2025). This creates compounded pressure: students experience financial difficulty alongside a heightened awareness of family responsibility.

Academic pressure intensifies this burden further. First-year students often face new academic expectations, unfamiliar assessment systems, and instruction in English, which may be their second or third language (Crookes, 2024). Some students in this study described lecturers and support staff as distant, which they attributed to differences in cultural background and communication norms on campus. Academic self-efficacy is closely linked to student wellbeing, with lower self-confidence associated with higher anxiety and disengagement (Naz et al., 2025; Upsher et al., 2025). Financial and academic pressures therefore function as interconnected sources of strain that compound one another during the first year, rather than as separate difficulties (Charlson et al., 2019; Naz et al., 2025).

### 2.6. Coping, Resilience, and Barriers to Effective Support

Research on coping strategies among university students identifies both problem-focused strategies, such as time management and help-seeking, and emotion-focused strategies, such as social support and spirituality (Cammock et al., 2021; Kowalenko et al., 2025). In Pacific Island contexts, coping is often relational and embedded in collective forms of support, including family, church, and village networks (Crookes, 2024; Xiong et al., 2025).

Relocation for study, however, can weaken access to these traditional support systems. Students may find themselves physically separated from the very networks that support their resilience. At the same time, help-seeking for mental health struggles is often shaped by stigma and cultural perceptions of psychological distress (Chang, 2011; Charlson et al., 2019). Formal counselling services may therefore be underused, not only because of stigma but also because they may not align with culturally familiar ways of understanding and discussing wellbeing (Crookes, 2021; Deva, 2014). This points to a gap between institutional support provision and students’ lived experience.

### 2.7. Theoretical Framework: Sensemaking and Student Experience

We are guided throughout by Weick’s (1995) theory of sensemaking, which explains how individuals interpret and construct meaning in uncertain and ambiguous situations. Sensemaking is grounded in identity, shaped by social interaction, and driven by plausibility rather than objective accuracy. It is triggered when existing meaning structures are disrupted (Weick, 1995).

This framework is particularly relevant to the first-year university transition, where students must interpret new academic, social, and cultural settings while also reconstructing their sense of self. Identity construction is central: students continually ask who they are and who they are expected to be within a new institutional setting. Sensemaking is also retrospective and social. Students construct meaning through reflection and interaction with others (Kowalenko et al., 2025; Upsher et al., 2025). These features align with Pacific relational worldviews, in which meaning is co-constructed within family and village contexts (Cammock et al., 2021; Crookes, 2024).

Sensemaking theory has been widely used in organisational studies (Weick, 1995), but its application to student mental health in Pacific Island higher education remains limited (Crookes, 2024; Lin et al., 2026). This study extends sensemaking theory into this empirical and cultural setting. We apply sensemaking as our single guiding framework throughout the analysis and discussion.

## 3. Methodology

### 3.1. Research Paradigm

We ground this study in the interpretive paradigm described by Burrell and Morgan (1979). The interpretive approach focuses on understanding social reality through the meanings people assign to their experiences. It assumes that reality is socially constructed and that knowledge is shaped by context and interpretation.

This approach fits the purpose of the study, which is to understand how students make sense of their mental health struggles during university transition. It allows attention to lived experience rather than external measurement, and it supports the view that students are active meaning-makers rather than passive subjects. This is particularly important in a Pacific Island context, where relational and cultural meanings shape experience (Cammock et al., 2021; Crookes, 2024). We recognise that norms related to age, gender, and family position are important in many Pacific communities, although their influence varies across the region’s diverse societies. Students, however, maintain agency in how they understand and negotiate their place within these social expectations. Relocating for study can disrupt this position and challenge the support networks connected to it.

### 3.2. Qualitative Research Design

We used a qualitative research design. Qualitative research focuses on meaning, process, and lived experience rather than precise measurement or prediction (Braun & Clarke, 2006, 2022). This design was appropriate because our aim was to explore how students understand and interpret their experiences.

A qualitative approach also allows attention to the emotional, cultural, and relational dimensions of mental health that are difficult to capture through quantitative methods. It provides the depth and detail needed to understand transition experiences in context (Braun & Clarke, 2022).

### 3.3. Participant Selection and Sampling

Seventeen first-year students from a regional university in Fiji participated in the study. Participants were recruited over a two-week period at the start of the second semester of the academic year, through the support of first-year course coordinators and the student support office. Students who expressed interest were screened against two inclusion criteria: enrolment in the first year of study, and completion of at least one full semester at the university. Recruitment continued until seventeen students were enrolled in three group interviews. Three additional students initially expressed interest but did not participate, mainly due to scheduling conflicts. Participants were selected using convenience sampling from students across the university’s twelve member countries. Three group interviews were held because participants indicated a preference for group discussion, which most said felt more comfortable than a one-to-one interview.

The sample included students from diverse Pacific Island backgrounds. All participants were enrolled in their first year and had completed at least one semester of study, which ensured they had sufficient experience to reflect on transition challenges. Table 1 summarises the composition of the sample and the three interview groups. Exact counts by country of origin are not reported, consistent with our confidentiality commitments in a small regional setting, but the sample spanned at least 7 of the university’s twelve member countries and included both male and female students across a range of study programmes.

Seventeen participants were considered sufficient for qualitative depth. The aim was not statistical representation but rich, diverse accounts of experience. Each participant is identified by a code such as P1 or P2 to maintain anonymity. Country of origin is not disclosed, to protect confidentiality in a small regional setting. Participants’ ages ranged from approximately 18 to 20 years. All were enrolled in the Bachelor of Commerce programme, with majors spanning Accounting, Finance, Economics, Management, Information Systems, and Human Resource Management. Individual ages and majors are not linked to specific participant quotes, to protect confidentiality and to prevent indirect identification within a small university community.

### 3.4. Data Collection: Semi-Structured Interviews

Data was collected through three semi-structured group interviews lasting between forty-five and seventy-five minutes. This method allowed flexibility while ensuring that key topics were covered across all interviews (Braun & Clarke, 2006). Groups ranged from five to six participants. Group composition was based on availability rather than pre-existing acquaintances, so participants did not necessarily know one another beforehand, although some overlap in social and residential networks was likely given the size of the university community. Each interview was facilitated by the second author, who has prior training and experience in qualitative interviewing and group facilitation. The second author is a Fiji Indian Muslim from Ba, Fiji. He later relocated to Suva for employment. He experienced challenges when adjusting to the urban environment of Suva. This experience provided him with an understanding of some of the challenges associated with relocating to Suva. His background also helped him understand the social and cultural context of the participants. However, he remained aware of his own experiences and assumptions during the interviews and analysis. Before each session, the facilitator explained the limits of group confidentiality. Participants were asked to treat one another’s disclosures as private.

An interview guide was used to explore arrival at university, emotional adjustment, academic experience, financial pressure, social relationships, and coping strategies. The guide was developed by the research team, drawing on the transition and Pacific higher education literature reviewed in Section 2, and was piloted informally with two students not included in the final sample before use. Questions were open-ended and followed by prompts to encourage deeper reflection.

Interviews were conducted in private spaces on campus. Participants were free to speak in English or in their preferred Pacific language, although English was used throughout in practice. All interviews were audio-recorded with consent and transcribed verbatim. The facilitator kept brief field notes after each session to record contextual observations, such as group dynamics and moments of visible distress, which informed subsequent analysis and debriefing.

### 3.5. Ethical Considerations

Participation was voluntary and based on informed consent. Participants were given clear information about the study and could withdraw at any time without consequence.

Written informed consent was obtained from all participants before participation. Participants received a participant information sheet and a consent form outlining the purpose of the study, procedures involved, potential risks and benefits, confidentiality measures, and their right to withdraw at any time without penalty. All participants signed the consent form before interviews began.

Confidentiality of individual identities was strictly maintained: recordings were stored securely, and transcripts were anonymised. Because data were collected in group interviews, however, we cannot guarantee confidentiality between participants themselves, and this limitation was explained to participants in advance, as noted in Section 3.4. Identifying details were removed from transcripts and quotations to protect participants, given the small and interconnected nature of the regional university context.

Because the topic involved mental health, the research team took specific steps to manage potential distress. Participants were told before each session that they could pause, decline any question, or stop the interview at any time without needing to give a reason. Contact details for the university’s student counselling and support services were provided to all participants in writing, both before and after each interview, together with a brief explanation of how to access these services. The facilitator monitored participants for visible signs of distress during interviews and was prepared to pause the discussion, offer a break, or end the session early if needed. However, this was not required in any of the three interviews conducted.

### 3.6. Reflexivity and Researcher Positioning

Reflexivity was central to the research process (Braun & Clarke, 2022). The first author is based at a university in the United Kingdom and has a long-standing research relationship with Fiji, without personal Pacific Islander heritage; the second author has direct institutional and cultural familiarity with the research context. The second author led recruitment, interview facilitation, and initial coding, while the first author contributed to formal analysis and the framing of the theoretical discussion. The researcher who had closer cultural and institutional relationship with participants also collected the data. This helped build rapport with participants and supported a deeper understanding of the local context. However, because of this close relationship, the research team reflected carefully during coding to avoid leaving shared assumptions unexamined.

We reflected regularly on our own assumptions and positionality throughout the study. We recognise that research interactions involve power relations that cannot be fully removed, given that one author held a role with some connection to student support functions at the university. Efforts were made to minimise this by allowing participants to lead the discussion and by treating their accounts as meaningful knowledge rather than simply as data.

### 3.7. Data Analysis: Thematic Analysis with NVivo

Data was analysed using reflexive thematic analysis following Braun and Clarke (2006, 2022). The process involved familiarisation with the data, systematic coding, theme development, review, and refinement. Coding was conducted in NVivo version 14. The second author completed first-pass line-by-line coding of all three transcripts. The first author independently reviewed the resulting coding structure and candidate themes, and disagreements in interpretation were resolved through discussion until agreement was reached. This process functioned as a reflexive check on interpretation rather than a test of inter-rater reliability in the positivist sense, consistent with Braun and Clarke’s (2022) approach to reflexive thematic analysis.

An inductive approach was used at the coding stage, so that initial themes emerged from the data rather than being imposed in advance. Sensemaking theory (Weick, 1995) was introduced only after inductive coding was complete, as an interpretive lens through which to further develop and discuss the six themes. It did not shape the initial coding frame. During this later interpretive stage, we also considered whether other theoretical lenses might illuminate specific extracts. We ultimately chose not to draw on these additional frameworks in the findings.

### 3.8. Trustworthiness

Trustworthiness was guided by the criteria of credibility, transferability, dependability, and confirmability (Lincoln & Guba, 1985). Credibility was strengthened through prolonged engagement with the data, the two-analyst coding and review process described in Section 3.7, and attention to alternative interpretations, including instances where participant accounts diverged from the dominant pattern within a theme; these are noted in Section 4.

Transferability was supported through detailed description of context and participants. Dependability was supported through an audit trail of analytic decisions. Confirmability was supported through reflexive practice and the use of direct participant quotations in the findings.

## 4. Findings and Discussion

Six thematic areas emerged from the analysis of interview data. These themes are presented and discussed below, with participant quotations used to ground the analysis in the texture of student experience. Findings are brought into dialogue with the existing literature and interpreted through the lens of Weick’s (1995) sensemaking theory. The themes are not mutually exclusive. They intersect and sometimes complicate one another, reflecting the relational character of Pacific student experience described by participants. Table 2 summarises the six themes. We report these themes as patterns of meaning within this specific sample of seventeen students, not as claims about prevalence across the wider Pacific student population; where participant accounts diverged from a theme’s dominant pattern, we note this explicitly in the relevant subsection below.

### 4.1. Navigating Homesickness and Emotional Separation

Nearly all participants described homesickness as among the most difficult aspects of their first year. One participant, P15, was an exception: she described her transition to Suva as comparatively smooth, attributing this to an older sibling already studying at the university who provided practical and emotional support from the outset. Her account suggests that pre-existing family networks on campus may buffer some of the difficulties described below. For most participants, however, what distinguished their accounts from the relatively straightforward descriptions of missing home found in some of the student adjustment literature was the depth and structural character of the separation they described. Participants did not simply miss their families. They described a sense of having been partly cut off from the relational systems through which their sense of self had been built and sustained.

*“I never thought it would be this hard. Back home, everything is around you, your parents, your brothers and sisters, your aunties, your church. When I came here, I kept waking up at night and not knowing where I was. It took me weeks before I felt like myself again, and even then, it was not the same self.”*

(P3)

This testimony resonates with Weick’s (1995) view of sensemaking as grounded in identity construction. The participant’s disorientation is not only emotional. It is a disruption to her basic sense of who she is. Without the relational infrastructure of home, the question of who one is becomes suddenly and disturbingly open. The student struggles to form a coherent identity while far from familiar sources of meaning and may become more vulnerable to others’ opinions or stereotypes. The phrase “not the same self” captures precisely the kind of identity disruption that, in sensemaking theory, triggers active sensemaking (Weick, 1995): a moment when existing frameworks for self-understanding can no longer be reliably applied.

Participants from smaller island nations who had come to Fiji for the first time described an additional layer of cultural displacement that compounded their homesickness. Several noted that the pace, noise, and social anonymity of Suva felt fundamentally alien to the rhythms of community life they had grown up within (Crookes, 2024; Lin et al., 2026).

*“In my home island everything moves slowly, and everyone knows everyone. Here you can walk past a hundred people and none of them look at you. That was very strange to me. I felt invisible in a way I had never felt before.”*

(P9)

The feeling of invisibility described here is significant. For several participants, especially those from smaller and more tightly connected island communities, social recognition felt closely tied to relationships with others (Cammock et al., 2021). The social anonymity of urban university life therefore represented, for these students, more than a change in social style. It disrupted a relational basis of personhood that required active sensemaking work to manage and partly resolve (Xiong et al., 2025). The existing literature on Pacific Island Communal Identity (Cammock et al., 2021; Crookes, 2024) supports this reading, though we emphasise that experiences of urban anonymity varied across participants and were described most acutely by those from smaller, more rural home communities.

Several participants described how communication technology, particularly social media and messaging platforms, provided a partial but limited compensation for physical distance. These same technologies could also intensify the sense of absence, particularly when participants witnessed family events or village celebrations from which they were absent (Xiong et al., 2025).

*“Seeing videos of my family celebrating my cousin’s birthday while I was sitting in my room eating noodles alone, that was one of the hardest moments of my year. I cried for a long time that night. Then I thought, okay, I have to study tomorrow, I have to keep going.”*

(P12)

The movement in this account from grief to resolve is characteristic of the resilience that many participants demonstrated. It also shows how sensemaking under emotional hardship often involves pragmatic reframing rather than a genuine resolution of the underlying difficulty. The student does not stop feeling the pain of exclusion. She sets it aside, provisionally, to meet the demands of academic survival. This is sensemaking at its most immediate and functional (Weick, 1995).

### 4.2. Making Sense of Financial Struggle and Survival

Financial hardship was a pervasive feature of participant accounts, shaping the quality of student life well beyond material inconvenience. Many participants received government or regional scholarships that did not fully cover the costs of accommodation, food, transport, and study materials in Suva. Others relied partly on remittances from family members who were themselves living with limited resources, which tied the student’s financial vulnerability directly to the financial circumstances of the wider family (Crookes, 2021; Naz et al., 2025).

*“My scholarship covers my fees and part of my accommodation. But for food and other things I must manage myself. Some weeks I eat one proper meal a day and make it work. I do not tell my parents because they are already sacrificing so much.”*

(P5)

The concealment of financial distress from family members, a pattern noted across several participant accounts, reflects a cultural logic in which the student’s role is understood as carrying the family’s hope for a better future (Xiong et al., 2024). Admitting to struggling is experienced not simply as personal disclosure but as a possible source of shame and added burden for those at home. This dynamic produces a form of silent suffering with real implications for mental health, since the students who most need support are often the least likely to seek it (Charlson et al., 2019; Crookes, 2021).

Research on financial stress and student wellbeing shows that material hardship significantly raises the risk of depression, anxiety, and academic disengagement (Naz et al., 2025; Xiong et al., 2025). In this study, these effects were compounded by the relational dimensions of financial obligation described above. Students were not only managing their own financial stress. They were also managing the emotional work of maintaining an acceptable face toward family and community, while privately dealing with hunger, exhaustion, and the difficulty of concentrating that ongoing financial anxiety produces (Crookes, 2024).

*“There are days when I cannot concentrate in class because I am thinking about how I will pay my rent next month. I sit there trying to understand the lecture and my head is somewhere else.”*

(P7)

From a sensemaking perspective (Weick, 1995), financial hardship functions as a continuous source of the uncertainty that triggers sensemaking. Students must constantly interpret their circumstances, adjust their spending, and build narratives that make their situation bearable enough to sustain continued engagement with their studies. This ongoing sensemaking work is demanding, and it competes directly with the demands of academic learning. Its absence from formal academic records or support referrals points to a real gap in institutional understanding of first-year student life for many students (Chang, 2011; Charlson et al., 2019).

### 4.3. Academic Pressure and Feelings of Inadequacy

A third theme across participant accounts was academic pressure and associated feelings of intellectual inadequacy. For many participants, the academic expectations of university study represented a marked step up from their secondary schooling, and this gap between prior preparation and current demand was experienced as psychologically destabilising (Crookes, 2024; Upsher et al., 2025).

*“At school I was always one of the top students. I thought university would be hard, but I would manage. Then in my first week I sat in a lecture, and I could not understand half of what the lecturer was saying. I thought, maybe I am not as smart as I thought. Maybe I do not belong here.”*

(P1)

The phrase “maybe I do not belong here” is significant. It suggests that academic difficulty was not experienced simply as a performance problem, but as a challenge to the student’s sense of having a rightful place within the institution. This is consistent with the literature on imposter syndrome among students from non-dominant backgrounds (Naz et al., 2025; Xiong et al., 2024). In this Pacific context, we suggest that this vulnerability may be influenced by students’ prior educational experiences, where they were often positioned as recipients of instruction rather than active participants in learning. However, our data does not allow us to establish this relationship with certainty. We therefore present this explanation as a plausible interpretation rather than a confirmed finding (Crookes, 2021, 2024).

Language posed a particular challenge for students whose primary language was not English. The university operates mainly through English-medium instruction, but students arrive with varying levels of English proficiency, and the gap between everyday spoken English and the formal academic English of lectures and assignments could be significant (Xiong et al., 2025).

*“I understand English, but when I read the assignments, I feel like they are written in a different language. The words are all English, but I cannot understand what they want me to do. I spent three hours on one paragraph once and I still was not sure if I was answering the question.”*

(P14)

This account illustrates a form of academic disorientation that is both linguistic and disciplinary. The student is not simply unfamiliar with vocabulary. She is grappling with an academic discourse that assumes familiarity with conventions of argument, citation, and disciplinary knowledge that her prior education has not equipped her with. From a sensemaking perspective (Weick, 1995), this is an encounter with a set of cues that is not yet legible to her.

Weick’s (1995) observation that sensemaking is driven by plausible rather than accurate interpretation is important here. Students who cannot fully decode academic expectations do not simply stop making sense. They generate plausible interpretations that allow them to act, even when those interpretations are imperfect. The key question for institutions is whether their support structures can refine these early, imperfect interpretations before academic disengagement becomes entrenched (Chang et al., 2015; Deva, 2014).

### 4.4. Reconstructing Identity in a New Environment

A fourth theme concerned identity disruption and reconstruction. Students described a process of renegotiating who they were in relation to their new social, cultural, and institutional context. This was not always experienced as straightforwardly negative: several participants described a gradual broadening of their sense of self as they encountered people and perspectives different from their own. For some, though, the process was genuinely destabilising (Crookes, 2024; Xiong et al., 2025).

*“Back home I knew exactly who I was. I was my parents’ daughter, I was from this place, I was part of this church. Here nobody knows any of that. Here I am just a student from a Pacific island, and I have to figure out what that means from scratch.”*

(P6)

This account captures the sense of identity stripping that can accompany the move to a university environment. In her home community, this participant’s identity was closely tied to specific relational and cultural memberships (Cammock et al., 2021). At university, she found herself grouped into the broader category of Pacific Island Students, a category that offers its own forms of community but does not carry the same specific relational meaning as her home identity. From a sensemaking perspective, she must now build a new, usable account of who she is within this less specified category, drawing on the resources available to her at university.

Encounters with other Pacific Island students were also, for some participants, a resource for identity reconstruction. Students who had initially experienced cultural displacement found in their relationships with peers from other island nations a shared Pacific identity that offered social comfort and a basis for meaningful connection (Crookes, 2024; Lin et al., 2026).

*“I became close friends with students from other islands. We are all Pacific; we understand each other’s struggles even if our islands are different. That community helped me feel less lost.”*

(P11)

This finding resonates with the literature on ethnic and cultural identity in higher education, which documents how students from minority or regionally specific backgrounds sometimes construct new forms of collective identity in response to shared experiences of displacement (Xiong et al., 2024). In the Pacific context, this process carries particular cultural weight: a shared Oceanic identity that extends beyond specific national affiliations has both cultural and political significance well beyond individual adjustment (Cammock et al., 2021). The emergence of this solidarity within the student body represents a form of collective sensemaking (Weick, 1995), through which a shared framework for interpreting the experience of dislocation is built together.

### 4.5. Social Isolation and the Search for Belonging

Social isolation was among the most commonly reported experiences across the seventeen participant accounts, and it operated at several levels: the absence of close family, the difficulty of making meaningful friendships in an academically competitive environment, the experience of feeling culturally marginal where certain national groups were numerically dominant, and the general loneliness of urban life (Crookes, 2021; Naz et al., 2025). For students accustomed to dense communal life in Pacific Island villages, the comparative social thinness of university interaction could feel deeply unsettling.

*“The first month I had nobody to eat lunch with. I would sit alone in the canteen and watch other students laughing with their friends and I would think, what is wrong with me? Why can I not just fit in?”*

(P2)

This account illustrates a pattern of self-blame that appeared in several participants’ narratives of social isolation. The student understood her isolation as a personal weakness rather than as a normal response to entering a new social environment. This interpretation reflects broader findings in the literature on social isolation (Gale & Parker, 2014). However, we cannot confirm this explanation because we did not collect data from staff perspectives. The accounts suggest that more relational engagement from lecturers and support staff may help students view isolation as a normal part of transition rather than as personal failure. We present this as a tentative implication of the findings rather than as a tested conclusion. This issue is discussed further as a recommendation in Section 5. It also suggests that unmediated isolation can be especially damaging when institutional structures fail to offer students alternative ways of understanding their experience (Charlson et al., 2019).

Several participants described the formal structures of university social life, such as clubs, societies, and orientation programmes, as inadequate to their needs. These structures felt superficial relative to the depth of connection students wanted (Crookes, 2024).

*“We had orientation week and there were activities and games. It was fun for a few days. But after that everybody went back to their own groups, and I was back to where I started. Those programmes do not go deep enough.”*

(P8)

This critique resonates with broader research on orientation and transition support in higher education, which notes that one-off activities are rarely sufficient to foster the sustained social integration first-year students need (Gale & Parker, 2014; Tinto, 1993). For at least some participants in this study, where belonging is relational and tied to specific cultural practices (Cammock et al., 2021), creating genuinely meaningful community within the university setting appears to require a more sustained and culturally attentive approach than current programmes offer (Chang et al., 2015; Deva, 2014).

### 4.6. Resilience, Adaptation, and Coping Strategies

Despite the pervasiveness and intensity of the difficulties described above, participant accounts also showed clear resilience, resourcefulness, and adaptive capacity. Students developed a range of strategies for managing the psychological and practical demands of first-year university life, drawing on personal, relational, cultural, and spiritual resources (Cammock et al., 2021; Crookes, 2024).

*“After a while I learned how to manage. I found a group of students who cooked together to save money. I found a church where I could sing on Sundays and feel something from home. Small things like that made a big difference.”*

(P4)

Building small, meaningful anchors of familiarity and community is a recognisable sensemaking strategy (Weick, 1995): the student is not resolving the structural conditions of her situation but is constructing stable reference points within it that allow her to keep functioning and making sense of her experience. Church featured in several participant accounts as a significant coping resource. This is consistent with the literature on the role of religious practice in Pacific Island community resilience (Cammock et al., 2021) and underlines the importance of spiritual and communal wellbeing frameworks for understanding Pacific student experience (Crookes, 2024).

Study groups and peer support relationships were also identified as important coping resources, particularly for academic challenges. Students who formed close academic relationships with peers, whether from their own island nation or elsewhere in the Pacific, reported feeling more confident and less isolated in their academic work (Xiong et al., 2025).

*“My study group saved me. We would meet three times a week and help each other understand the readings and prepare for assessments. Knowing I did not have to face it alone changed everything.”*

(P16)

This account illustrates the social dimension of sensemaking clearly (Weick, 1995). The student’s improved ability to make sense of her academic environment is not simply a product of individual effort. It reflects a relational context in which interpretation is negotiated and refined together. The study group functions as a small sensemaking community, in which the uncertainty of academic demands is gradually reduced through shared interpretive work (Kowalenko et al., 2025).

Several participants also described gradual adaptation as a form of narrative reframing, in which the first year, looking back, came to be understood as a period of growth rather than simply a period of suffering (Xiong et al., 2024).

*“Looking back now, I can see that first year made me stronger. I did not see it at the time. At the time I just wanted to go home. But now I know I can handle things I never thought I could.”*

(P17)

This retrospective reframing is, in Weick’s (1995) terms, close to the essence of sensemaking: the construction of a coherent, usable account of struggle that allows the individual to act effectively in the present and future. The narrative of strength and growth this student builds from her first-year experience does not deny the difficulties she faced. It is a sensemaking achievement, one that turns what might otherwise remain a story of suffering into a foundation for continued engagement in academic life.

## 5. Conclusions

This study examined how first-year students at a regional university in Fiji make sense of their mental health struggles during the transition into university life. Based on interviews with seventeen students from different Pacific Island nations, the findings show that first-year student life is shaped by a combination of emotional, social, financial, academic, and identity-related challenges. These challenges were often experienced together, and often without adequate support (Charlson et al., 2019; Crookes, 2021).

Six main themes were identified: homesickness and emotional separation, financial struggle, academic pressure, identity reconstruction, social isolation, and resilience. These themes are closely connected rather than separate. Students who faced financial hardship and food insecurity also tended to report homesickness, identity confusion, and academic stress in the same accounts. This overlap points to cumulative pressure on mental wellbeing within this sample. This is consistent with earlier work showing that student transition is academic as well as social and emotional (Gale & Parker, 2014; Xiong et al., 2025). Our findings suggest that these pressures may be particularly intense for Pacific Island students who move far from home and have limited institutional support (Chang, 2011; Crookes, 2024). However, this study cannot determine whether these experiences are significantly different from those of other student populations.

Theoretically, the study extends Weick’s (1995) sensemaking theory by applying it to Pacific Island student mental health in higher education. The findings show that students actively construct meaning around their experiences through identity work, reflection, and social interaction. Sensemaking, in this sample, was shaped by relationships and cultural expectations: collective identity and family obligation played an important role in how students interpreted stress and hardship, and spiritual and cultural values acted as important anchors for meaning making (Cammock et al., 2021; Crookes, 2024). This extends sensemaking theory by showing, within this specific setting, how it can operate in culturally grounded ways.

The study also contributes to Pacific higher education research by focusing on lived student experience at a regional university in Fiji. Previous research has highlighted structural barriers in Pacific higher education but has often overlooked the personal and emotional dimensions of student life (Chang et al., 2015; Hunter, 2012; Lin et al., 2026). This study addresses that gap by showing how students describe and interpret university life in their own words, and how these interpretations are shaped by both structural conditions and cultural expectations (Deva, 2014; Naz et al., 2025).

In practical terms, the findings point to several tentative implications, which we offer as directions for institutional consideration rather than as prescriptive conclusions, given the scope of this study. First, mental health services may benefit from being more culturally responsive, so that students are not required to engage only with clinical models that do not reflect Pacific ways of understanding wellbeing (Chang, 2011; Charlson et al., 2019). Second, financial hardship emerged as a major source of strain in this sample; reviewing scholarship levels, accommodation support, and emergency assistance against real living costs may help address this (Crookes, 2021; Xiong et al., 2025). Without such review, some students may continue to spend much of their first year managing basic needs, which our data suggest can affect both learning and wellbeing (Crookes, 2024; Naz et al., 2025). Third, peer support and mentoring programmes that are sustained over time, rather than limited to short orientation periods, may better reflect Pacific values of collective responsibility and belonging (Cammock et al., 2021; Kowalenko et al., 2025). Fourth, and offered cautiously given the limits of our data on staff perspectives, participant accounts of social isolation and self-blame (Section 4.5) suggest that more relational engagement from lecturers and support staff, alongside professional standards, could help some students interpret early difficulties as a normal part of transition rather than as personal failure.

### 5.1. Limitations

This study has several limitations. First, the sample included seventeen students and was appropriate for qualitative depth (Braun & Clarke, 2006). However, participants were recruited through convenience sampling at a single institution, which limits statistical generalisation to the wider Pacific Island student population. Second, the cross-sectional design captured student experiences at one point in time and cannot show how sensemaking develops across the full academic year. Third, although participants could use their preferred Pacific language, interviews were conducted in English in practice. This may have influenced how some participants expressed emotionally difficult experiences. Fourth, both researchers were affiliated with the higher education sector, and one researcher had an institutional connection to the research site. Therefore, we cannot completely exclude the influence of shared professional assumptions on interpretation, despite the reflexive practices described in Section 3.6.

### 5.2. Future Research

Future research should use longitudinal designs to track how student sensemaking develops over time. Comparative studies across different campuses in the regional university system would also be useful (Lin et al., 2026). More focused research is needed on female students and students from smaller island nations (Xiong et al., 2024). Future studies should examine staff perspectives directly rather than relying only on student accounts to understand implications for staff practice. Further research should also test culturally adapted mental health and peer support programmes in Pacific higher education settings (Charlson et al., 2019; Deva, 2014).

## Figures and Tables

**Table 1:** Summary of participants and interview groups.

Interview Group	No. of Participants	Approx. Countries Represented	Gender Composition	Interview Length
Group 1	6	4	4 males 2 females	62 min
Group 2	6	5	3 males 3 females	50 min
Group 3	5	3	1 male 4 females	72 min
Total	17	7	8 males 9 females	45–75 min range

Source: Created by authors.

**Table 2:** Overview of themes.

Theme	Brief Description	Illustrative Participants
Homesickness and emotional separation	Disruption to relational identity and belonging caused by distance from family and home community; one partial exception noted (P15).	P3, P9, P12, P15
Financial struggle and survival	Material hardship compounded by concealment of difficulty from family and moral obligation to succeed.	P5, P7
Academic pressure and feelings of inadequacy	Gap between prior preparation and university demands, intensified by academic English.	P1, P14
Identity reconstruction	Renegotiation of self within a broader, less specific “Pacific Island student” category.	P6, P11
Social isolation and belonging	Loneliness and self-blame; formal orientation activities described as insufficient.	P2, P8
Resilience and coping	Use of relational, spiritual, and peer resources; retrospective narrative reframing.	P4, P16, P17

Source: Created by authors.

## Data Availability

The interview data are not publicly available because they contain information that could identify participants within a small regional university community, and participants did not consent to public data sharing. De-identified excerpts beyond those reported in this article may be made available by the corresponding author on reasonable request, subject to institutional approval.
